# Complete Genome and Plasmid Sequences of *Staphylococcus aureus* EDCC 5055 (DSM 28763), Used To Study Implant-Associated Infections

**DOI:** 10.1128/genomeA.01698-16

**Published:** 2017-02-23

**Authors:** Gopala Krishna Mannala, Torsten Hain, Cathrin Spröer, Boyke Bunk, Jörg Overmann, Volker Alt, Eugen Domann

**Affiliations:** a Laboratory of Experimental Trauma Surgery, Justus-Liebig University Giessen, Giessen, Germany; b Institute of Medical Microbiology, Justus-Liebig University Giessen, Giessen, Germany; c German Center for Infection Research (DZIF), Partner Site Giessen-Marburg-Langen, Giessen, Germany; d Leibniz Institute, German Collection of Microorganisms and Cell Cultures (DSMZ), Braunschweig, Germany; e German Center for Infection Research, (DZIF), Partner Site Hannover-Braunschweig, Braunschweig, Germany; f Department of Trauma Surgery, Justus-Liebig-University and University Hospital Giessen, Giessen, Germany

## Abstract

*Staphylococcus aureus* EDCC 5055 (DSM 28763) is a human clinical wound isolate intensively used to study implant-associated infections in rabbit and rat infection models. Here, we report its complete genome sequence (2,794,437 bp) along with that of one plasmid (27,437 bp). This strain belongs to sequence type 8 and contains a *mecA* gene.

## GENOME ANNOUNCEMENT

Staphylococci occur ubiquitously in nature and are colonizers of skin and mucous membranes of mammals and birds. *Staphylococcus aureus* is considered to be the most important human pathogen among the staphylococci. Approximately 30% of the human population carries this pathogen asymptomatically in the anterior nares. However, *S. aureus* becomes an opportunistic pathogen when the host defense system is weakened, and it is considered a major nosocomial pathogen involved in soft skin infections, causing severe diseases comprising sepsis, endocarditis, toxic shock syndrome, and osteomyelitis (1). The clinical use of antibiotics for treatment of *S. aureus* infections has resulted in a rise in strains with antimicrobial resistance (2). *S. aureus* EDCC 5055 (Eugen Domann Culture Collection number 5055) deposited in German Collection of Microorganisms and Cell Cultures (DSM number 28763) was isolated from a human wound infection in 1995 and has been used as a model pathogen to study implant-associated infections (3–8). We determined the complete genome sequence of this strain using Illumina and PacBio sequencing platforms.

For whole-genome sequencing, genomic DNA was isolated from an overnight culture grown at 37°C in brain heart infusion (BHI) medium using the Qiagen Genomic-tip 100/G DNA extraction kit. Sequencing was performed with Illumina MiSeq (Illumina, The Netherlands) and PacBio RSII (Pacific Biosciences, USA) sequencers. For Illumina sequencing, a library was constructed using the Nextera XT sample prep kit. Paired-end sequencing yielded a total of 5,450,074 paired reads with a mean read length of 101 bp. P6-C4 chemistry was used for PacBio sequencing, which generated 150,292 prefiltered reads, with an average read length of 4,699 bp.

*De novo* genome assembly was carried out based on 48,617 postfiltered PacBio reads, with an average read length of 8,637 bp under the “RS_HGAP_Assembly.3” protocol included in SMRT Portal version 2.3.0. Finally, end trimming and circularization were performed. The chromosome was adjusted to *dnaA* as the first gene, and annotation was performed using Prokka 1.11 (9).

The complete closed chromosome of *S. aureus* EDCC 5055 (DSM 28763) comprises 2,794,437 bp and has a G+C content of 32.84%, 2,549 coding sequences, 62 tRNAs, and 19 rRNAs. One intact prophage region was identified using PHAST. The strain also harbors a plasmid (termed pEDCC_5055) with a size of 27,437 bp.

*In silico* analysis revealed that the strain belongs to the sequence type 8 (ST8) and *spa* type 24 (t024). Its genome includes multiple chromosomal and plasmid-carried virulence genes (*sak*, *scn*, *splA*, *splB*, *splE*, *aur*, *lukD*, *lukE*, *hlb*, *hlgA*, *hlgB*, and *hlgC*), enterotoxins (*ser*, *sej*, *sed*, and *sea*), and antimicrobial resistance genes (*spc*, *mecA*, *norA*, and *ermA*) (10–12). *S. aureus* EDCC 5055 (DSM 28763) appeared as a cryptic methicillin-resistant *S. aureus* (MRSA) strain (*mecA* gene present but not expressed), since the strain showed phenotypically no multiresistance at the beginning of isolation. Over the years of usage, the *mecA* gene became expressed and the MRSA phenotype became apparent (13). The high-quality closed genome sequence of *S. aureus* EDCC 5055 (DSM 28763) will provide the basis for future comparative genomics, for the understanding of the emergence of antimicrobial resistance, and in the study of host-pathogen interactions using transcriptomics.

### Accession number(s).

This whole-genome shotgun project has been deposited in the European Nucleotide Archive under the accession numbers LT671859 (complete chromosome sequence) and LT671860 (complete plasmid sequence). The versions described in this paper are the first versions.

## References

[1] Becker K, Skov RL, von Eiff C 2015 *Staphylococcus*, *Micrococcus*, and other catalase-positive cocci, p 354–382 *In* Jorgensen JH, Pfaller MA, Carroll KC, Funke G, Landry ML, Richter SS, Warnock DW (ed), Manual of clinical microbiology, 11th ed, vol 1. ASM Press, Washington, DC.

[2] Chambers HF, Deleo FR 2009 Waves of resistance: *Staphylococcus aureus* in the antibiotic era. Nat Rev Microbiol 7:629–641. doi:10.1038/nrmicro2200. 19680247 PMC2871281

[3] Alt V, Bitschnau A, Österling J, Sewing A, Meyer C, Kraus R, Meissner SA, Wenisch S, Domann E, Schnettler R 2006 The effects of combined gentamicin-hydroxyapatite coating for cementless joint prostheses on the reduction of infection rates in a rabbit infection prophylaxis model. Biomaterials 27:4627–4634. doi:10.1016/j.biomaterials.2006.04.035. 16712926

[4] Alt V, Lips KS, Henkenbehrens C, Muhrer D, Oliveira Cavalcanti MC, Cavalcanti-Garcia M, Sommer U, Thormann U, Szalay G, Heiss C, Pavlidis T, Domann E, Schnettler R 2011 A new animal model for implant-related infected non-unions after intramedullary fixation of the tibia in rats with fluorescent *in situ* hybridization of bacteria in bone infection. Bone 48:1146–1153. doi:10.1016/j.bone.2011.01.018. 21281750

[5] Alt V, Kirchhof K, Seim F, Hrubesch I, Lips KS, Mannel H, Domann E, Schnettler R 2014 Rifampicin-fosfomycin coating for cementless endoprostheses: antimicrobial effects against methicillin-sensitive *Staphylococcus aureus* (MSSA) and methicillin-resistant *Staphylococcus aureus* (MRSA). Acta Biomater 10:4518–4524. doi:10.1016/j.actbio.2014.06.013. 24948548

[6] Mohamed W, Sommer U, Sethi S, Domann E, Thormann U, Schütz I, Lips KS, Chakraborty T, Schnettler R, Alt V 2014 Intracellular proliferation of *S. aureus* in osteoblasts and effects of rifampicin and gentamicin on *S. aureus* intracellular proliferation and survival. Eur Cells Mater 28:258–268. doi:10.22203/eCM.v028a18. 25340805

[7] Sethi S, Thormann U, Sommer U, Stötzel S, Mohamed W, Schnettler R, Domann E, Chakraborty T, Alt V 2015 Impact of prophylactic CpG oligodeoxynucleotide application on implant-associated *Staphylococcus aureus* bone infection. Bone 78:194–202. doi:10.1016/j.bone.2015.04.030. 25959416

[8] Mohamed W, Domann E, Chakraborty T, Mannala G, Lips KS, Heiss C, Schnettler R, Alt V 2016 TLR9 mediates *S. aureus* killing inside osteoblasts via induction of oxidative stress. BMC Microbiol 16:230. doi:10.1186/s12866-016-0855-8. 27716055 PMC5048406

[9] Seemann T 2014 Prokka: rapid prokaryotic genome annotation. Bioinformatics 30:2068–2069. doi:10.1093/bioinformatics/btu153. 24642063

[10] Angiuoli SV, Gussman A, Klimke W, Cochrane G, Field D, Garrity GM, Kodira CD, Kyrpides N, Madupu R, Markowitz V, Tatusova T, Thomson N, White O 2008 Toward an online repository of standard operating procedures (SOPs) for (meta)genomic annotation. Omics 12:137–141. doi:10.1089/omi.2008.0017. 18416670 PMC3196215

[11] Zankari E, Hasman H, Cosentino S, Vestergaard M, Rasmussen S, Lund O, Aarestrup FM, Larsen MV 2012 Identification of acquired antimicrobial resistance genes. J Antimicrob Chemother 67:2640–2644. doi:10.1093/jac/dks261. 22782487 PMC3468078

[12] Joensen KG, Scheutz F, Lund O, Hasman H, Kaas RS, Nielsen EM, Aarestrup FM 2014 Real-time whole-genome sequencing for routine typing, surveillance, and outbreak detection of verotoxigenic *Escherichia coli*. J Clin Microbiol 52:1501–1510. doi:10.1128/JCM.03617-13. 24574290 PMC3993690

[13] Domann E, Hossain H, Füssle R, Chakraborty T 2000 Rapid and reliable detection of multiresistant *Staphylococcus aureus* (MRSA) by multiplex PCR. Dtsch Med Wochenschr 125:613–618. doi:10.1055/s-2007-1024385. 11256043

